# Correspondence

**Published:** 1868-09

**Authors:** Josiah Bacon

**Affiliations:** Treasurer.


### ARTICLE VIII.

Dr. F. J. S. Gorgas,

*Ed. Am. Journal Dental Science, Baltimore, Md.*

Dear Sir.

In your July number I have had called to my notice, an article regarding certain suits of this Company, *vs* Drs. Smith of Syracuse, Hamess of Skaneateles, and Matson and Tripp of Auburn, for the use of the so called "Porter" rubber. The notice states that Injunctions were refused in these cases. This statement is untrue. Drs. Smith and Hamess, are both Licensees of this Company, and long since settled all our claims against them. No Injunction has been asked for against them and none of course refused. The statement is an injury to these gentlemen. Against Tripp and Matson we had suits for infringement committed since January 1st. —both these parties however made oath that they *had not made a single plate of rubber since January 1st.* whereupon Judge Hall in *their* case, very properly denied our motion for Injunction.

Since that date ( June 21) however, affidavits and samples of work done by one of these parties in *April or May* last have been laid before the court, and Dr. Tripp was enjoined at the court sitting at Buffalo July 9th. Such parties are clearly liable to a further charge the proof of which will be laid before the U. S. District Attorney.

The false statements regarding these suits are made by the "Porter Company" manufacturers of the so called " Simpson" or "Porter" rubber, to induce dentists to purchase, their rubber under the impression they can use it with impunity. I regret that your Journal should thus spread the fraud. I send you a copy of the opinion of the Courts on this question, and stand ever ready to give you *truthful* information. Please correct your statement, and oblige.

Yours Very Respectfully,

JOSIAH BACON, Treasurer.

